# Biogeochemical Signals from Deep Microbial Life in Terrestrial Crust

**DOI:** 10.1371/journal.pone.0113063

**Published:** 2014-12-17

**Authors:** Yohey Suzuki, Uta Konno, Akari Fukuda, Daisuke D. Komatsu, Akinari Hirota, Katsuaki Watanabe, Yoko Togo, Noritoshi Morikawa, Hiroki Hagiwara, Daisuke Aosai, Teruki Iwatsuki, Urumu Tsunogai, Seiya Nagao, Kazumasa Ito, Takashi Mizuno

**Affiliations:** 1 Graduate School of Science, The University of Tokyo, 7-3-1 Hongo Bunkyo-ku, Tokyo 113-0033, Japan; 2 Geological Survey of Japan, Advanced Industrial Science and Technology, Higashi 1-1-1, Tsukuba, Ibaraki 305-8567, Japan; 3 Graduate School of Environmental Studies, Nagoya University, Furo-cho, Chikusa-ku, Nagoya 464-8601, Japan; 4 Japan Atomic Energy Agency, 1-64 Yamanouchi, Akiyo-cho, Mizunami, Gifu 509-6132, Japan; 5 Low Level Radioactivity Laboratory, Kanazawa University, 24, Wake, Nomi, Ishikawa 923-1224, Japan; Missouri University of Science and Technology, United States of America

## Abstract

In contrast to the deep subseafloor biosphere, a volumetrically vast and stable habitat for microbial life in the terrestrial crust remains poorly explored. For the long-term sustainability of a crustal biome, high-energy fluxes derived from hydrothermal circulation and water radiolysis in uranium-enriched rocks are seemingly essential. However, the crustal habitability depending on a low supply of energy is unknown. We present multi-isotopic evidence of microbially mediated sulfate reduction in a granitic aquifer, a representative of the terrestrial crust habitat. Deep meteoric groundwater was collected from underground boreholes drilled into Cretaceous Toki granite (central Japan). A large sulfur isotopic fractionation of 20–60‰ diagnostic to microbial sulfate reduction is associated with the investigated groundwater containing sulfate below 0.2 mM. In contrast, a small carbon isotopic fractionation (<30‰) is not indicative of methanogenesis. Except for 2011, the concentrations of H_2_ ranged mostly from 1 to 5 nM, which is also consistent with an aquifer where a terminal electron accepting process is dominantly controlled by ongoing sulfate reduction. High isotopic ratios of mantle-derived ^3^He relative to radiogenic ^4^He in groundwater and the flux of H_2_ along adjacent faults suggest that, in addition to low concentrations of organic matter (<70 µM), H_2_ from deeper sources might partly fuel metabolic activities. Our results demonstrate that the deep biosphere in the terrestrial crust is metabolically active and playing a crucial role in the formation of reducing groundwater even under low-energy fluxes.

## Introduction

Based on recent updates from scientific ocean drilling [1], it is estimated that the terrestrial subsurface potentially harbours the largest prokaryotic biomass on Earth [2]. Oceanic crust is generally less than 0.2 Gyr old because of subduction processes resulting from plate tectonics [3], whereas terrestrial crust has served as a vast and stable habitat for microbial life throughout Earth's history. The terrestrial deep biosphere has been intensively studied for the Kalahari craton, which is associated with a productive gold mine in South Africa [4]. This 4-km deep microbial ecosystem is colonized by microbial species energetically dependent on H_2_ and sulfate mainly produced by water radiolysis in radionuclide-enriched Precambrian formations. Because H_2_ production by low temperature rock-water interactions is generally slow, hydrothermal circulation is thought to be essential for the support of H_2_-dependent microbial populations in the oceanic and terrestrial crusts [5], [6].

Granite is the most abundant rock in the terrestrial crust with a maximum age of 40 Ga [7]. As for the deep granitic biosphere, H_2_–dependent activities of microbial reductions of sulfate and CO_2_ have been found in the Scandinavian shield [8], where the last deglaciation triggered a sea level rise and seawater intrusion into the deep aquifer. It is therefore suggested that the source of H_2_ originated from microbial fermentation of photosynthetically derived organic compounds recently introduced into the aquifer. However, the majority of the deep terrestrial crust is characterized by the percolation of recharged meteoric water through granitic rocks and the eventual depletion of photosynthetically derived organic matter and oxidants. Despite the importance of the deep terrestrial crust in this regard, the existence of microbial life and the mode of metabolic activity remain largely unknown [9].

**Figure 1:**
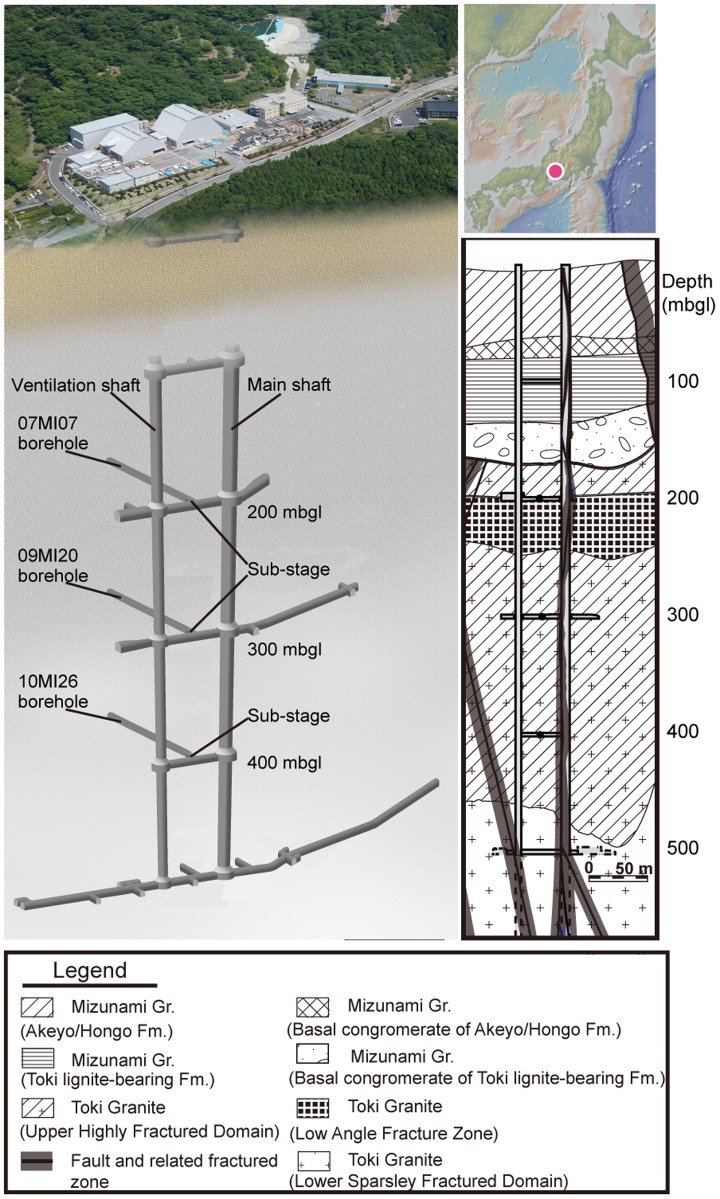
The location and schematic underground layout of the Mizunami Underground Research Laboratory (MIU) with investigated boreholes and a geological cross section^14^. Each legend aspect in the geological cross-section represents a lithological unit or rock fracture property differences.

Biogeochemical processes mediated in a freshwater-dominated granitic aquifer were investigated at the Mizunami Underground Research Laboratory (MIU), currently being constructed in Gifu Prefecture, central Japan (Fig. 1). The MIU comprises a main shaft, a ventilation shaft, and sub-stages placed every 100 m with depth. Horizontal boreholes 07MI07, 09MI20 and 10MI26 were drilled from sub-stages at 200, 300 and 400 m below ground level (mbgl), respectively (Fig. 1). Near these boreholes, several vertical faults intersect the granitic basement (Fig. 1) [10]. Over the past six years, the groundwater chemistry was periodically measured for the concentrations and isotopic compositions of dissolved chemicals.

## Materials and Methods

### Study site

Around the MIU construction site, Tertiary sedimentary rocks of the Akeyo, Hongo, and Toki lignite-bearing formations (∼100–200 mbgl) overlie Cretaceous Toki granite [11], [12] (Fig. 1). Near the MIU site, Tono uranium ore deposits disseminated within the Toki lignite-bearing formation have been investigated for the long-term migration of uranium [13]. Geologic, hydrologic and hydrochemical properties of the MIU site have been previously characterized ([11] and reference therein). By using the vertical boreholes, the groundwater chemistry around the MIU site was determined to be Na-(Ca)-Cl type in the lower sedimentary formation and the granitic basement [11]. The boreholes were sectioned by a multi-packer system into six intervals with interval #6 closest to the borehole orifice. The use of cement to seal the borehole orifices inserted with the multi-packer system caused high pH groundwater enriched with Ca. However, hydraulic connectivity between the interval #6 and the other adjacent intervals was low, and geochemical data showed no evidence for groundwater mixing between the intervals. The Japan Atomic Energy Agency granted field permission

### Sampling

Groundwater sampling began immediately after the packer installation: 07MI07 borehole in October 2007, 09MI20 borehole in December 2009 and 10MI26 borehole in January 2011. Groundwater data (December 2012) were compiled for this study. It should be noted that the hydraulic pressures of all the investigated borehole intervals sharply increased following to Great East Japan Earthquake (March 11, 2011) [14]. Because all boreholes were artesian, groundwater was directly collected without air exposure via plastic tubing connected to each interval. Before sampling, groundwater was drained extensively (at least three interval volumes) to ensure that groundwater from the geological formation around the borehole was sampled. The second interval from 10MI26 was not investigated because of a lack of groundwater outflow.

For the analysis of dissolved oxygen (DO), groundwater was collected directly into an evacuated glass ampule pre-treated with an indigo carmine solution (Low Range Dissolved Oxygen AccuVac Ampuls; HACH, Loveland, CO, USA). For the analysis of groundwater isotopic composition (*δ*D_H_2__O__ and *δ* ^18^O_H_2__O__), groundwater was collected into a 1L polycarbonate bottle after rinsing with sample groundwater three times. For the analysis of ion concentrations, including Na^+^, K^+^, Ca^2+^, Mg^2+^, Cl^−^, NO_3_^−^, NO_2_^−^, SO_4_^2−^, NH_4_^+^, Fe^2+^ and HS^−^, groundwater was flushed for>8 h through a double-ended stainless bottle (1L) equipped with needle-type stop valves (Swagelok Co., Ltd., Solon, OH, USA). After flushing, the valves were closed, groundwater collected and immediately analysed. For organic acid analysis, including acetate, fumarate, formate, lactate, succinate, malate, pyruvate and citrate, a subsample was filtered through a filter (0.22 µm pore size, type GVWP; Millipore, Billerica, MA, USA) with polypropylene housing, collected into a 50-ml polypropylene bottle (BD Falcon, Becton, Dickinson company, Franklin Lakes, NJ, USA) that was rinsed three times with groundwater, and then stored at 4°C until analysis. For the analysis of dissolved organic carbon (DOC) and humic substances, subsamples were filtered through a pre-combusted (450°C for 4 h) 0.45-µm glass fibre filter (GF/F; Whatman, Buckinghamshire, UK), collected in pre-combusted 30-ml glass vials with screw caps and frozen until analysis.

For the analysis of dissolved H_2_, groundwater was collected directly into a pre-evacuated glass vial (65 ml volume, Nichiden-Rika Glass Co., Ltd., Hyogo, Japan) sealed with a butyl grey rubber stopper through a disposable micro-syringe needle and then immediately analysed on site. For the analysis of CH_4_ and C_2_H_6_, and the carbon isotopic composition of CH_4_, sampling was the same as for H_2_ and followed by the addition of degassed HgCl_2_-saturated solution. For analysis of sulfur isotopic compositions of SO_4_^2−^ and HS^−^, groundwater was collected through a membrane filter (pore size 0.22 µm, type GVWP; Millipore, Billerica, MA, USA) into a 1L polycarbonate bottle after rinsing three times. Then, 5 ml of zinc acetate (2N) and 0.5 ml of NaOH (5N) were added and the solution was stored at room temperature. Dissolved noble gases were directly collected without air exposure in annealed copper tubes.

### Geochemical analyses

The pH and temperature were measured in a flow-through cell by multi-parameter electrodes and probes (Ocean Seven 305; Idronaut, Brugherio, Italy). Measurement precision for pH and temperature was ±0.1 and ±0.1°C, respectively. The concentration of dissolved oxygen (DO) was measured by the Indigo Carmine method with an absorptiometer (DR2800, HACH, Loveland, CO, USA) at a precision of ±0.006 mg/L. The isotopic composition of the groundwater (*δ*D_H_2__O__ and *δ* ^18^O_H_2__O__) was determined by isotope ratio mass spectrometry (IsoPrime; Isoprime Ltd, Cheadle, UK), following the reduction of H_2_O to H_2_ and equilibration of H_2_O and CO_2_. The precision was ±1‰ for *δ*D_H_2__O__ and ±0.1‰ for *δ* ^18^O_H_2__O__.

For the following analyses, a subsample was filtered through a 0.45-µm membrane filter with polypropylene housing. The concentrations of ionic species Na^+^, K^+^, Ca^2+^, Mg^2+^, NH_4_^+^, Cl^−^, NO_3_^−^, NO_2_^−^, SO_4_^2−^ and PO_4_^3−^ were analysed with ion chromatography (ICS-1000; Dionex Corp., Sunnyvale, CA, USA). The analytical precision was ±10%. By using ferrous iron reagent powder pillows and hydrogen sulfide reagents (HACH, Loveland, CO, USA), the Fe^2+^ and HS^−^ concentrations were measured colourimetrically with a spectrophotometer (UVmini-1240; Shimadzu Co., Kyoto, Japan) with a precision of ±10%. Concentrations of dissolved organic carbon (DOC) and dissolved inorganic carbon (DIC) were measured by a combustion carbon analyser (TOC-_VCSH_; Shimadzu Corp.). The low level of DOC (<70 µM) in 2011–2012 was quantified by thermocatalytic oxidation and MC-NDIR detection (multi N/C 3100; Analytik Jena Japan, Kanagawa, Japan). The organic acid concentrations (acetate, fumarate, formate, lactate, succinate, malate, pyruvate and citrate) were analysed by a high performance liquid chromatograph coupled to a conductivity detector (Prominence, Shimadzu Co., Kyoto, Japan). The precision of the organic acid analysis was ±10%.

The concentration of H_2_ was measured with a trace reduction gas detector (TRA-1; Round Science, Kyoto, Japan) based on the mercury oxide to mercury vapour conversion [15]. The TRA-1 consists of a pre-cut column (5 cm long, 1 mm i.d.) and a main column (50 cm long, 2.18 mm i.d.) that is packed with a molecular sieve (5 Å) to separate H_2_ from CO and other gases. The eluted H_2_ was transferred by H_2_-free air into an analytical cell, where the concentration was determined by an ultraviolet spectrometer. The total error, incorporating the sampling and analysis, was ±20%.

Humic substances were separated from the other organic matter and analysed by the high performance size exclusion chromatography (HPSEC) system previously described [16]. The groundwater sample (100 µl) was injected into the HPSEC column with a 20 sample loop at a carrier flow rate of 1 ml/min. The mobile phase was a 0.01 M Tris-HCl buffer solution with 0.01 M NaCl and an adjusted pH of 8.0. Fluorescence was monitored at an excitation wavelength of 320 nm and an emission wavelength of 430 nm as a function of elution volume. Similar to granitic groundwater from the Tono uranium mine [16], groundwater from the MIU site was associated with three fluorescence peaks. Total areas of the fluorescence peaks obtained at a range of elution volume from 8 to 13 ml were subdivided by a factor of 10000. This method does not determine the absolute concentrations of humic substances, but the quantitative comparison among groundwater samples allowed temporal and spatial monitoring of humic substance changes based on the fluorescence peaks.

The concentrations of CH_4_ and C_2_H_6_ and their carbon isotopic compositions (*δ* ^13^C_CH_4__ and *δ* ^13^C_C_2___H_6__) were determined using a Finnigan MAT 252 isotope ratio mass spectrometer (Thermo Finnigan, Bremen, Germany) as described previously [17]. To determine CH_4_ and C_2_H_6_ concentrations and *δ* ^13^C_CH_4__, an analytical system consisting of a CO_2_-trapping port with Ascarite II and a liquid O_2_ temperature trap was used. The concentration and carbon isotope precision were ±5% and ±0.2‰, respectively. The hydrogen isotopic composition of CH_4_ (*δ*D_CH_4__) was determined using a CF-IRMS system (Trace GC and Delta-V, Thermo Fisher Scientific, Bremen, Germany). To determine the *δ*D_CH_4__ values, purified CH_4_ was used with a ceramic tube unit (35 cm long, 1 mm i.d.) held at 1350°C to thermally decompose CH_4_ to H_2_ and C. The analytical precision of the *δ*D of CH_4_ was better than 10‰.

DIC, including HCO_3_^−^ and CO_3_^2−^, in the groundwater was precipitated as SrCO_3_ by adding ammonia-concentrated SrCl_2_ solution to water samples in the field. The precipitated SrCO_3_ was converted to CO_2_ through a reaction with phosphoric acid in a vacuum line. The carbon isotopic composition of DIC (*δ* ^13^C_DIC_) was determined using an isotope ratio mass spectrometer (Micromass Dual inlet OPTIMA, Waters Corporation, Milford, MA, USA). The sulfur isotopic compositions of SO_4_^2−^ and HS^−^ (*δ* ^34^S_SO_4___^2−^_ and *δ* ^34^S_HS^−^_, respectively) were analysed by an elemental analyser-isotope ratio mass spectrometer (DELTA^plus^ Advantage ConFlo III System, Thermo Fisher Scientific, Bremen, Germany). The stored water sample was filtered to collect ZnS. While the filter, intended for sulfide analysis, was dried in a freeze dryer overnight, the filtrate, which was for sulfate analysis, was combined with 1N BaCl_2_ to precipitate BaSO_4_, then dried overnight in a freeze dryer. The filter samples were packed into tin containers and introduced into a Flash EA1110 (Thermo Fisher Scientific, Bremen, Germany) where they were transformed into SO_2_. The precision of the sulfur isotope analysis was ±0.3‰.

The concentrations of He and Ne and He isotopic composition were measured with a noble gas mass spectrometer (Micromass MM5400, Waters Corporation, Milford, MA, USA) located at the Geological Survey of Japan. Technical details of the dissolved noble gas extraction and mass spectrometry, including the purification procedures, were described previously [18]. The reproducibility of the ^3^He/^4^He ratio as well as the He and Ne concentrations obtained from replicated measurements of air saturated water (ASW) were about 2%, 3% and 3%, respectively.

### Electron microscopy

In 2011, suspended particles in groundwater were collected on 0.22-µm pore size, 25-mm diameter polycarbonate filter (Advantec, Tokyo, Japan). The filter was dehydrated with 100% ethanol and dried in a desiccator. The portion of the filter was coated with Pt-Pd and observed by SEM by using a Hitachi S4500 equipped with energy dispersive X-ray (EDX) spectroscopy at 5-kV and 15-kV accelerating voltages for secondary electron imaging and EDX analysis, respectively.

### Thermodynamic calculations

Saturation indices of calcite (CaCO_3_) and gypsum (CaSO_4_·2H_2_O) in groundwater samples were calculated by PHREEQ C [19]: 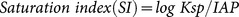
where Ksp is the solubility product of a mineral and IAP is the ion activity product. When SI  =  0, the groundwater is in equilibrium with a mineral. When SI>0 and SI <0, a mineral precipitates and dissolves, respectively.

*In situ* Gibbs reaction energy (*ΔG*) of hydrogen-oxidizing sulfate reduction: 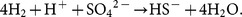
was calculated using the following equation:
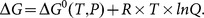
where Δ*G^0^(T, P)* is the Gibbs standard reaction energy at *in situ* temperature and pressure, *R* is the ideal gas constant, *T* is groundwater temperature, and *Q* denotes the activity of the reactants and reaction products. The *ΔG^0^(T, P)* was calculated using the SUPCRT92 software package [20]. The activities of reactants and products were calculated using the Geochemist's Workbench software package [21].

### Mass balance equation for groundwater mixing and microbial consumption of sulfate

To calculate the amount of microbially reduced sulfate in the granitic aquifer, a mass balance equation was derived that incorporates the sulfate dilution by mixing of shallow and deep groundwater and the removal of sulfate by microbial reduction: 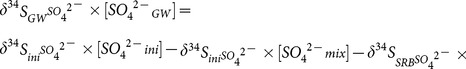
(1)where *δ* ^34^S_ini_^SO_4_^ ^2−^ and [SO_4_^2−^ini] denote the *δ* ^34^S value and concentration of sulfate initially recharged to the granitic aquifer, [SO_4_^2−^mix] is the sulfate concentration diluted by groundwater mixing, *δ* ^34^S_SRB_^SO_4_^ ^2^ and [SO_4_^2−^SBR] are the *δ* ^34^S value and concentration of sulfate removed by microbial sulfate reduction, and *δ* ^34^SGW^SO_4_^ ^2−^ and [SO_4_^2−^GW] are the *δ* ^34^S value and concentration of sulfate measured in groundwater samples. For the calculation, it was assumed that deep groundwater is completely depleted in sulfate, based on data from Iwatsuki et al. [11], and that groundwater mixing is not accompanied by sulfur isotopic fractionation. Because groundwater flow is inferred to be slow from the residence time of groundwater [22], the mass balance equation was developed as a closed system.

Because [SO_4_^2−^mix] can be expressed as: 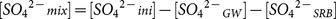
(2)


Replacing [SO_4_^2−^mix] in Eq. (1) with [SO_4_^2−^ini] − SO_4_^2−^GW] − [SO_4_^2−^SRB] gives:
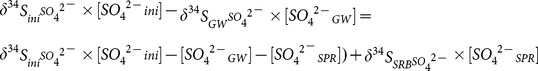
(3)


The expansion of Eq. (3) is:
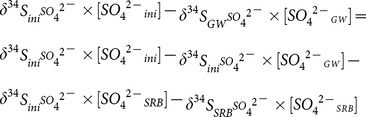
(4)


Elimination of *δ* ^34^S_ini_ SO_4_^2−^ × [SO_4_^2−^ini] results in: 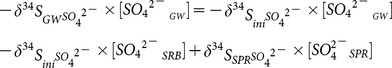
(5)


Integrating common factors gives: 
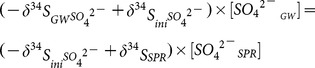
(6)


Thus, [SO_4_^2−^SRB] is calculated as:
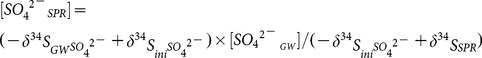
(7)


*δ* ^34^S_SRB_ seems to be calculated:
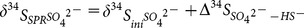
(8)


However, the shift in *Δ* ^34^SSO_4_^2−^−HS^−^ from the initial state to the present state was taken into account. *Δ* ^34^SSO_4_^2−^−HS^−^ was linearly correlated with sulfate concentration (R^2^  =  0.703): 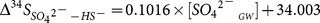
(9)


To derive *δ* ^34^S_SRB_ SO_4_^2−^, *Δ* ^34^S_ini→_GWSO_4_^2−^−HS^−^ was calculated: 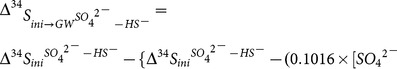
(10)where a *Δ* ^34^S_ini_ SO_4_^2−^−HS^−^ value and [SO_4_^2−^ini] were set to be 52.29‰ and 180 µM, respectively.

*δ* ^34^S_SRB_ SO_4_^2−^ was finally calculated: 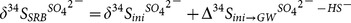
(11)


*δ* ^34^S_ini_ SO_4_^2−^ was assumed to be 10‰, based on data from Iwatsuki et al. [12]

## Results and Discussion

### Hydrogeochemcal disturbance of the granitic aquifer

**Figure 2:**
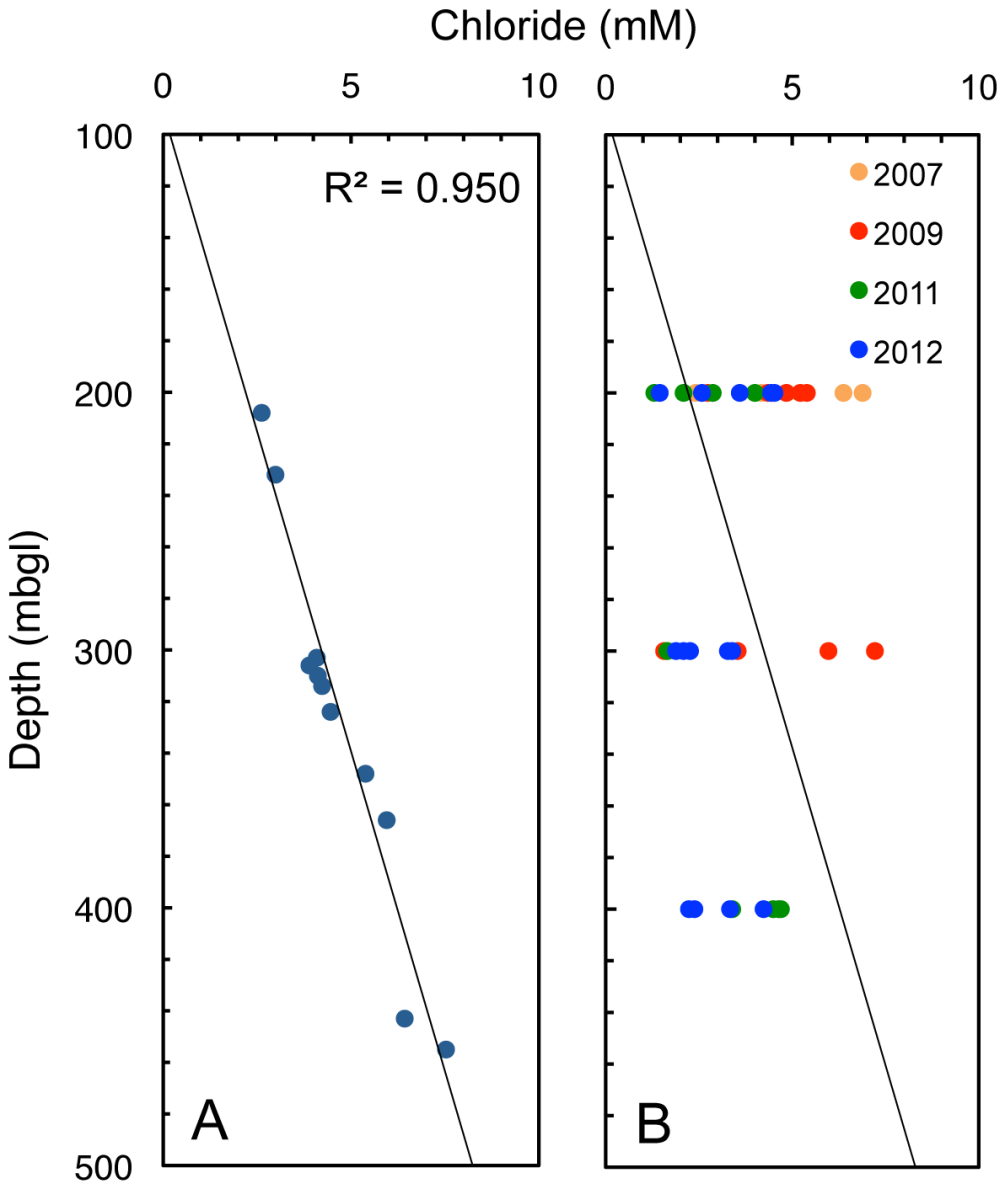
The correlation of depth with chloride in the groundwater before and during shaft construction. (A) Pre-excavation correlation from DH-2 borehole, which was drilled ∼100 m south of the ventilation shaft before the shaft construction. (B) Post-excavation correlation from underground boreholes. Each different circle colour represents annual differences in sampling date. A straight line was fitted through the data by least squares and regression analysis. The deviation from linearity is expressed as a correlation coefficient (R^2^).

As evident from *δ*D_H2O_ and *δ* ^18^O_H2O_ values (S1 Table in S1 File), seawater has been flushed by meteoric water at great depth since 5 Ma [11]. Based on vertical borehole investigations prior to the shaft construction [11], the original depth profiles of dissolved chemicals have been determined. Chloride linearly increases with depth (R^2^ = 0.950, Fig. 2A), whereas sulfate and dissolved inorganic carbon (DIC) linearly decrease with depth (R^2^ = 0.664 and 0.843, respectively). The concentrations of sulfate and DIC are similarly correlated with chloride concentration (R^2^ = 0.659 and 0.802, respectively). From the linear depth profiles of chloride, sulfate and DIC, it is inferred that shallow groundwater with low chloride and high sulfate and DIC infiltrated deep groundwater with high chloride and low sulfate and DIC. After the shaft excavation, the original depth profile of chloride (Fig. 2B) was dramatically changed by the upward movement of deeper groundwater [10].

Previously, extensive groundwater mixing has been recognized during the construction of underground shafts in granitic aquifers in Canadian and Fennoscandian Shields [23], [24]. Sampling of deep groundwater originally distributed in the granitic aquifer seems to be technically impossible where hydraulic conductivity is high due to high facture connectivity. In contrast, sampling of deep granitic groundwater from a borehole drilled from the surface is not suitable for accurate determination of the concentrations of redox sensitive species, because of the extensive intrusion of oxygenated drilling fluid into the aquifer. Because horizontal boreholes 07MI07, 09MI20 and 10MI26 were drilled mostly with outflowing groundwater instead of artificial drilling fluid, drilling disturbance was minimized. Additionally, groundwater sampling for gaseous species was conducted at substages under *in situ* hydraulic pressure conditions, which could avoid degassing during pumping groundwater from the surface.

**Figure 3:**
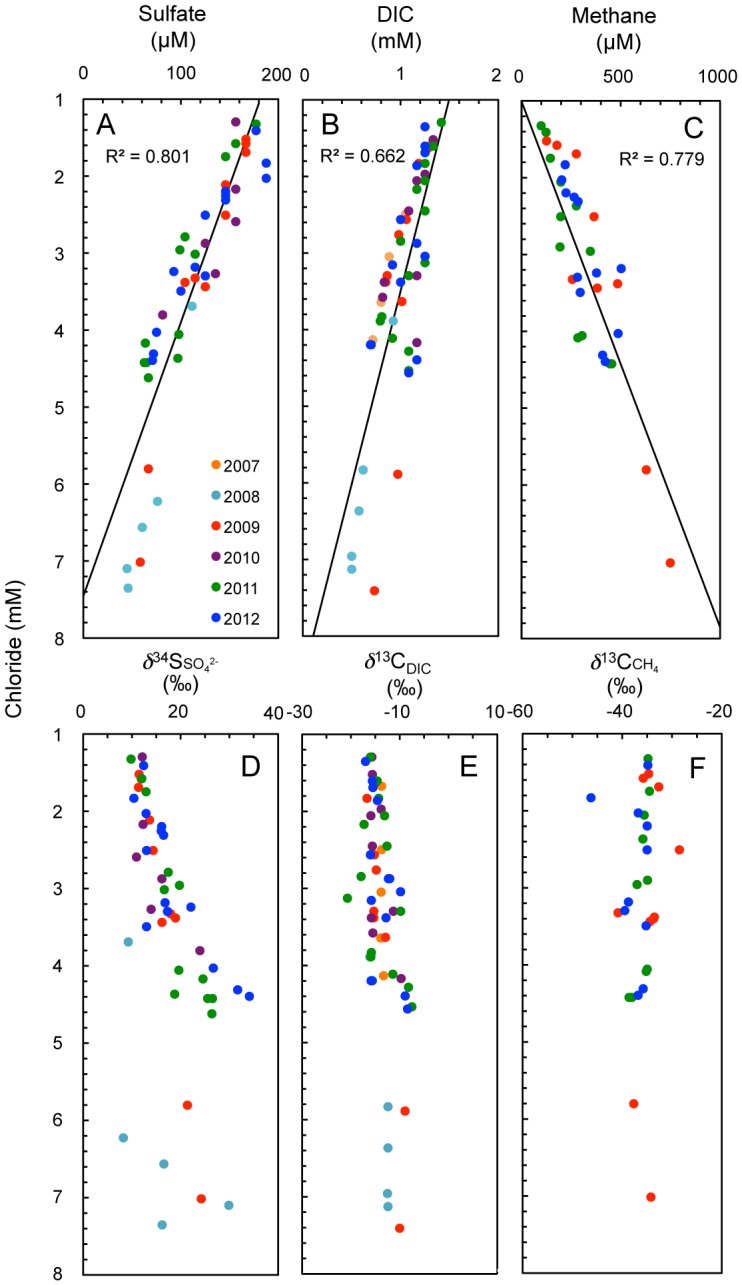
Correlations of chloride concentrations with biologically metabolized compounds and their isotopic compositions: (A) sulfate concentrations, (B) concentrations of dissolved inorganic carbon (DIC), (C) methane concentrations, (D) *δ* ^34^SSO_4_^2−^ values, (E) *δ* ^13^C_DIC_ values and (F) *δ* ^13^C_CH4_ values. Each different circle colour represents annual differences in sampling date. A straight line was fitted for all obtained data by least squares and regression analysis. The deviation from linearity is expressed as a correlation coefficient (R^2^). Isotope standards for *δ* ^13^C and *δ* ^34^S values were Vienna Pee Dee Belemnite and Cañon Diablo meteorite, respectively.

By taking the advantages and disadvantages of groundwater sampling from the underground facility into account, it was attempted to reconstruct the original biogeochemical profiles by long-term monitoring of the concentrations of aqueous and gaseous species. Despite the hydrologic disturbance (S1 Figure in S1 File), linear correlations with chloride were maintained for sulfate (R^2^ = 0.801, Fig. 3A) and DIC (R^2^ = 0.662, Fig. 3B) over the monitoring periods. Methane (CH_4_) also linearly increased with chloride (R^2^ = 0.779, Fig. 3C). Because chloride is well known as a conservative tracer in groundwater [25] and had clear correlation with depth before shaft excavation, the depth profiles of sulfate, DIC and CH_4_ were reconstructed based on the corresponding chloride concentrations.

### Biogeochemical processes controlling sulfate, DIC and CH_4_ profiles

All groundwater samples were near equilibrium with calcite (CaCO_3_). Because near-equilibrium of calcite has been maintained prior to the shaft construction [11], it is not likely that calcite precipitation was promoted by groundwater mixing. Additionally, all groundwater were highly undersaturated with respect to gypsum (CaSO_4_·2H_2_O) (S1 Table in S1 File). Hence, the decreases in the sulfate and DIC concentrations with depth were not attributed to mineral precipitation reactions.

To clarify the influences of microbiologically mediated processes on the depth trends of sulfate, DIC and CH_4_, carbon and sulfur isotopic compositions were analysed (Figs. 3D–F, S3 Table in S1 File). By plotting the *δ* ^34^S values of sulfate, ^34^S enrichment with increasing chloride concentration was found (Fig. 3D) that indicates the involvement of microbial sulfate reduction [26]. In contrast, the *δ* ^13^C values of DIC and CH_4_ were relatively constant over a wide range of chloride concentrations (Figs. 3E–F), which indicates mixing of shallow and deep groundwater with contrasting DIC and CH_4_ concentrations. Because large negative and positive *δ* ^13^C shifts of CH_4_ were not evident, it is likely that microbial production and consumption of CH_4_ were not substantial enough to overprint the simple mixing profile [27]. Because acetate was not detected in the groundwater (S2 Table in S1 File), microbial reduction of CO_2_ to acetate might be negligible.

**Figure 4:**
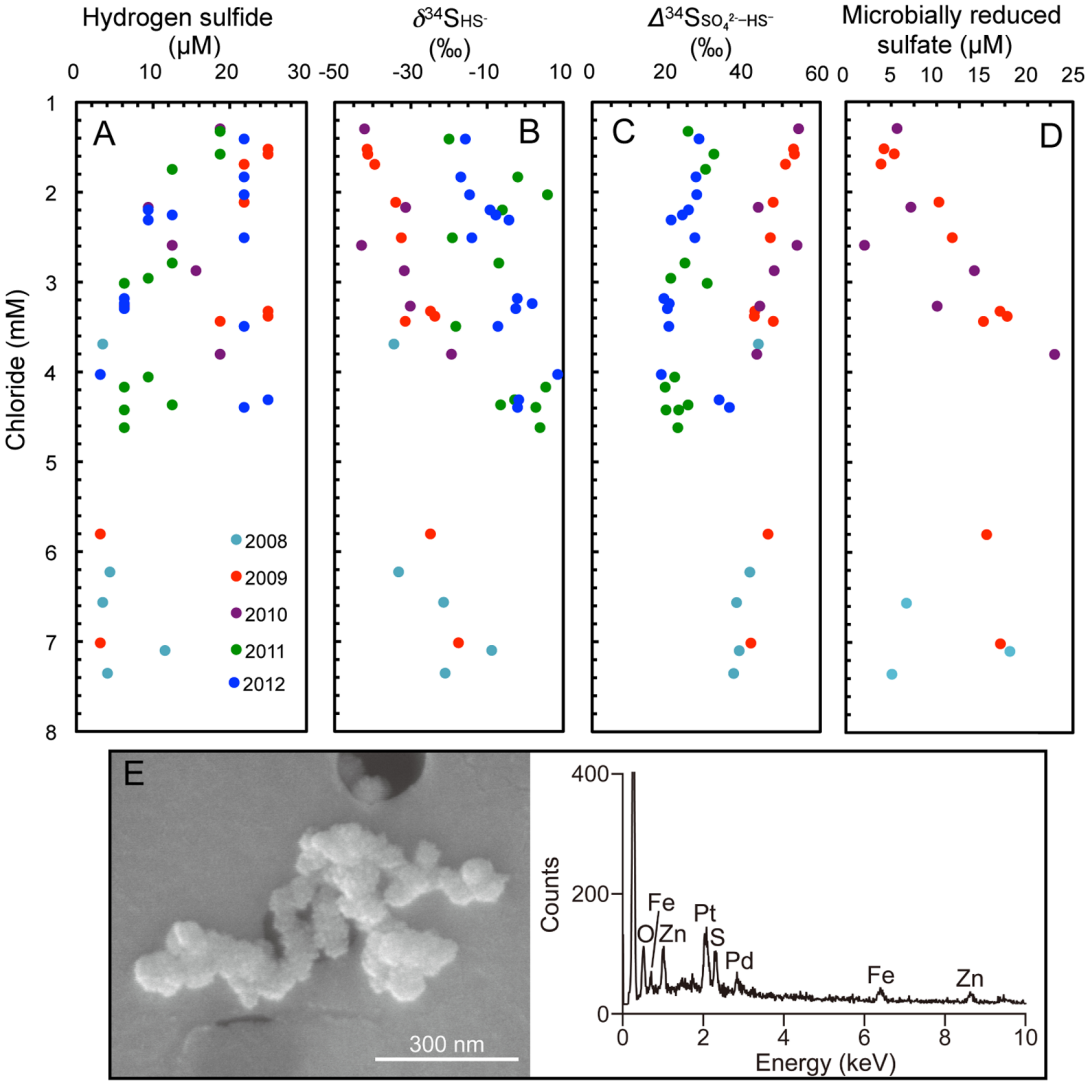
Correlations of chloride concentrations with (A) hydrogen sulfide concentrations, (B) *δ* ^34^S_S^2−^_ values, (C) *Δ* ^34^S_SO_4_^2−^_ _-HS^−^_ values, and (D) estimated amounts of microbially reduced sulfate and (E) secondary electron image and EDX spectrum of suspended particles in groundwater from the #4 borehole of 09MI20 borehole. Estimated amounts from 2011–2012 are not included because of the abrupt shifts in *δ* ^34^S_S^2−^_ and *Δ* ^34^S_SO_4_^2−^_ _-HS^−^_values between 2008–2010 and 2011–2012. The shifts might be caused by groundwater mixing after shaft construction. Each different circle colour represents annual differences in sampling data.

It is well established that in low temperature aqueous systems, microbial reduction is a dominant process for the removal of sulfate, accompanied by the preferential consumption of isotopically light sulfate and the production of isotopically light hydrogen sulfide [28]. In contrast to sulfate, hydrogen sulfide concentrations were not linearly correlated with chloride (Fig. 4A). Mineral particles suspended in groundwater were collected by filtration and observed by SEM equipped with EDX analysis. Groundwater from #4 interval of 09MI20 borehole. This interval was selected because sulfide concentrations were relatively low over the monitoring period. As a result, mineral particles composed of Zn, Fe and S were detected (Fig. 4E), which suggests that the lack of linearity was caused by the precipitation of metal sulfides in the aquifer. Microbial mediation of sulfate reduction coupled to sulfide production is strongly suggested by ^34^S depletion in sulfide relative to sulfate (Figs. 4B–C, S3 Table in S1 File), although the *δ* ^34^S fractionation was significantly reduced after sampling in 2011. This abrupt shift in sulfur isotopic fractionation might have been caused by microbial sulfate reduction influenced by recent groundwater mixing and the following stabilization of groundwater (S1 Figure in S1 File) [10]. Two reaction pathways for large sulfur isotopic fractionation are known: microbial sulfate reduction and sulfur disproportionation [26]. Although sulfur disproportionation may have proceeded under micro-aerobic conditions caused by drilling disturbance, the sulfur disproportionation fractionation results in ^34^S-enriched sulfide and ^34^S-depleted sulfate [26]. Because sulfate reduction is associated with larger sulfur isotope fractionation between sulfide and sulfate than sulfur disproportionation, the decreases in fractionation after 2011 are counterintuitive because sulfur disproportionation should diminish and be succeeded by sulfate reduction.

**Figure 5:**
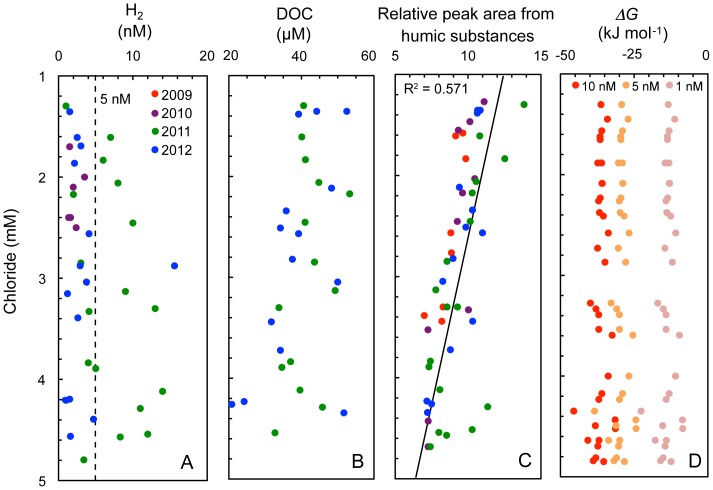
Correlations of chloride concentrations with potential energy sources and available energy from H_2_-oxidizing sulfate reduction: (A) H_2_ concentrations, (B) the concentrations of dissolved organic carbon, (C) relative peak areas from humic substances semi-quantified by high-performance size exclusion chromatography, (D) calculated Gibbs energy yields from H_2_-oxidizing sulfate reduction at *in situ* temperature and pressure at H_2_ concentrations found in the granitic aquifer. Each different circle colour represents annual differences in sampling date. A straight line was fitted for all obtained data by least squares and regression analysis. The deviation from linearity is expressed as a correlation coefficient (R^2^).

H_2_ is a central electron donor for microbial energy generation and not easily transported in groundwater because of continuous cycling by microorganisms [29]. Thermodynamic calculations and laboratory- and field-based measurements consistently report that terminal electron accepting processes (TEAPs) have different affinities to H_2_ uptake: <0.1 nM for O_2_ and nitrate reduction, 0.1–1 nM for Fe(III) reduction, 1–5 nM for sulfate reduction, and 5–30 nM for CO_2_ reduction [30]. H_2_ is therefore considered a sensitive indicator for an *in situ* TEAP occurring in groundwater. Along with the sulfur isotopic profiles in the aquifer, most H_2_ concentrations were within a range characteristic for sulfate reduction (Fig. 5A, S2 Table in S1 File). Although an increase in H_2_ concentration was temporarily observed in 2011, H_2_ concentrations dropped to previous levels in the next year.

### Potential energy sources for sulfate reduction

Organic carbon typically serves as an energy source for microbial sulfate reduction. The concentration of dissolved organic carbon (DOC) was barely detectable and not correlated with chloride (Fig. 5B). In contrast, highly sensitive measurements, established for humic substances derived from surface vegetation and overlying sedimentary rocks in the study area, revealed a clear negative correlation with chloride (Fig. 5C; R^2^ = 0.571). As humic substances are less available for metabolic activities, it is reasonable that humic substances decrease with depth by groundwater mixing similarly to sulfate and DIC. If sulfate reducers use organic carbon for energy and carbon sources, the concentration of DOC is supposed to decrease with depth. The lack of correlation with chloride might be explained by authigenic production of DOC. The negligible *δ* ^13^C shift of DIC with chloride might be explained by the small amount of authigenic organic carbon isotopically depleted in ^13^C compared with the DIC pool of groundwater. Because it is also known that fermentation of organic matter produces acetate in the deep terrestrial subsurface [6], there is the possibility that the low acetate levels could be minor energy and carbon sources for sulfate-reducing microorganisms.

Alternative electron donors available in the sulfate-reducing aquifer are CH_4_ and H_2_, It is known that anaerobic oxidation of methane is suppressed at sulfate concentrations below 0.5 mM [31] and/or at H_2_ concentrations above 0.1 nM [32]. Therefore, H_2_ best serves as an alternative electron donor in the granitic aquifer. *In situ* Gibbs energy of H_2_–oxidizing sulfate reduction was calculated from the activities of products and reactants. The Gibbs energy yield at 1 nM H_2_ was −13.6±2.7 kJ mol^−1^ (Fig. 5D), which is substantially lower than −23±1.2 kJ mol^−1^ reported as the minimum Gibbs energy required for H_2_–oxidizing sulfate reduction in anoxic sediments [30]. These lower values might reflect an optimization of energy-conserving efficiency by indigenous microorganisms in the H_2_–limited granitic aquifer. The Gibbs energy yield of −29.5±2.7 kJ mol^−1^ at 5 nM H_2_ appears to be sufficient to conserve energy by H_2_–dependent sulfate reduction.

Interestingly, an intensive H_2_ flux has been detected in a granitic cataclasite outcrop on an active fault 33 km northeast of the site [33]. Because a vertical fault intersects the underground MIU facility (Fig. 1), the fractures associated with the fault might serve as a fast conduit for deeply produced H_2_. As previously reported from the surrounding area [34], isotopic analyses of noble gases revealed high ratios of mantle-derived ^3^He relative to radiogenic ^4^He in groundwater from the monitoring boreholes (S4 Table and S2 Figure in S1 File). Thus, H_2_ generated in the mantle by high temperature reactions among dissolved gases in the C–H–O–S system [35] might be supplied along with ^3^He to the granitic aquifer.

### Hydrogeochemical estimation of in situ rate of sulfate reduction

The amount of microbially reduced sulfate in the granitic aquifer was quantified by solving a mass balance equation. For the calculations, it was assumed that initial sulfate (*δ* ^34^S_iniSO_4_^2−^_) with a value of 10‰ is mixed with deep sulfate-depleted groundwater without sulfur isotopic fractionation. It was also assumed that microbial reduction results in the removal of sulfate so the extent of ^34^S fractionation in groundwater was defined as *Δ* ^34^S_ini→GWSO_4_^2−^_−HS^−^_. Data after sampling in 2011 were excluded because *Δ*^34^S_SO_4_^2−^__−HS^−^_ values were sustantially shifted (Fig. 4D), possibly by the effects of shaft excavation and groundwater mixing on metabolic activities. The amount of microbially reduced sulfate ranged from ∼5–25 µM (S5 Table in S1 File and Fig. 4E), which is roughly consistent with the range of HS^−^ concentrations (Fig 4A). The sulfur isotopic signatures of sulfate and hydrogen sulfide along the groundwater flow path are often modelled by Rayliegh fractionation [4], because the deep aquifer can be treated as a closed system due to mass transport limitation. As *Δ*^34^S_SO_4_^2−^__−HS^−^_ was varied with sulfate concentration, Rayliegh fractionation model that requires the constant fractionation was not applicable to this investigated aquifer._

Groundwater from the lower unit of the overlying sedimentary formation has been previously estimated by ^14^C content to have a residence time of 9.3 ka [11]. ^4^He age of granitic groundwater was measured to be ∼10 ka between recharge and middle areas in the study area [22]. ^4^He age of discharge area where the MIU is located was difficult to determine, because groundwater is associated with high ^3^He/^4^He ratio and enriched with ^4^He (S2 Figure in S1 File). From the age estimates based on ^14^C and ^4^He, it is therefore assumed that the residence time of groundwater in the discharge area is, at least, ∼10 ka. If the amount of microbially reduced sulfate (5–25 µM) was consumed for ∼10 ka, the *in situ* rate of microbial sulfate reduction is estimated to be 1–5 nM yr^−1^. This rate range is slightly higher than those from well-characterized deep biospheres, such as the diverse microbial community in sub-seafloor sediments (0.66 nM yr^−1^) [36] and the microbial ecosystem at the South African Gold Mine (0.22−1.45 nM yr^−1^) [4].

### The existence of low-energy crustal biosphere

Temperature and the concentrations of electron donors and sulfate are known to affect the kinetics of sulfur isotope fractionation in microbial sulfate reduction because sulfate transport across cell membranes and enzymatic transformations of sulfate to sulfide are both rate-limiting [26]. The sulfur isotopic fractionation is generally considered to be less than 10‰ at the low sulfate levels (<0.2 mM) based on a lack of fractionation demonstrated in a variety of laboratory culture conditions [37]. It is also well known that microbial respiration rates in nature are overestimated by 3–6 orders of magnitude because *in situ* conditions are difficult to reproduce in laboratory-based activity measurements [9], [38]. When a laboratory culture is limited by H_2_ supply at sulfate concentrations of ∼0.2 mM, a large fractionation is observed and correlated with a reduced rate of sulfate reduction [39]. Because the *in situ* rate of sulfate reduction and the concentration of H_2_ appear to be substantially lower in the granitic aquifer than those manipulated in the laboratory, large fractionation values appear reasonable under extreme energy limitation. Indeed, very large sulfur isotopic fractionation in low sulfate groundwater affected by sulfate reduction has been known for a long time in deep sedimentary aquifers [40], [41]. The mechanism of this large fractionation remains to be shown; the kinetics of enzymatic activities from sulfate to sulfide might be slower than that of sulfate transport across cell membrane due to low-energy fluxes [26].

The existence of a H_2_-based deep biosphere has been previously reported from groundwater in the Columbia River Basalt. However, the production of H_2_ by low-temperature interactions between basalt and water is controversial with respect to the long-term sustainability of subsurface lithotrophic microbial ecosystems (SLiMEs) [42]. Terrestrial and deep-sea hydrothermal fluids are colonized by thermophilic and hyperthermophilic methanogenic hydrogenotrophs, suggesting that hydrothermal activity is generally essential for the production and supply of H_2_ [4]–[6]. The deep biosphere unveiled in this study is unique in that the crustal habitat is supplied with low fluxes of energy sources without hydrothermal circulation. Our findings have significant implications for the disposal of nuclear wastes because microbes can establish reducing conditions under low-energy fluxes, which would substantially decrease the mobility of many radionuclides [43].

## Supporting Information

S1 File **S1 Figure**, Changes in chloride concentration during shaft construction: (A) 07MI07 borehole, (B) 09MI20 borehole, (C) 10MI26 borehole. Each circle colour represents different sampling intervals. **S2 Figure**, Correlation between ^3^He/^4^He and ^4^He/^20^Ne in deep groundwater samples from boreholes drilled from the surface in the Tono area (black circles) [22] and from the MIU sub-stages (blue circles). The solid lines delineate the mixing lines between air saturated water (ASW) and the upper mantle, and between ASW and the crust. **S1 Table**, Temperature, isotopic composition and pH of groundwater samples and the concentrations of major cations and anions as well as saturation indices (SI) of calcium carbonate and sulphate minerals. VSMOW  =  Vienna Standard Mean Ocean Water. **S2 Table**, Groundwater concentrations of biologically utilized compounds. **S3 Table**, Carbon, hydrogen and sulphur isotopic composition of biologically metabolized compounds in groundwater. VPDB and CDT indicate Vienna Pee Dee Belemnite and Cañon Diablo meteorite, respectively. **S4 Table**, Groundwater concentrations and isotopic composition of noble gases. **S5 Table**, Estimated amounts of microbially reduced sulphate in the granitic aquifer.
(DOCX)
