# Supplementary material for: Biogeochemical Signals from Deep Microbial Life in Terrestrial Crust
Source: PLoS One. 2014 Dec 17;9(12):e113063. doi: 10.1371/journal.pone.0113063 (PMC4269445; doi:10.1371/journal.pone.0113063)

**Supplementary Information**

Table S1. Temperature, isotopic composition and pH of groundwater samples and the concentrations of major cations and anions as well as saturation indices (SI) of calcium carbonate and sulphate minerals. VSMOW = Vienna Standard Mean Ocean Water.

|  | 07MI07-1 | 07MI07-2 | 07MI07-3 | 07MI07-4 | 07MI07-5 | 09MI20-1 | 09MI20-2 | 09MI20-3 | 09MI20-4 | 09MI20-5 | 10MI26-1 | 10MI26-3 | 10MI26-4 | 10MI26-5 |
| --- | --- | --- | --- | --- | --- | --- | --- | --- | --- | --- | --- | --- | --- | --- |
| Temp. (^o^C) | 8.8 | 9.5 | 10 | 9.6 | 8.5 | 20 | 20.3 | 20 | 16.9 | 20.2 | 22.6 | 21 | 24.2 | 23.3 |
| *δ*DH_2_O (‰VSMOW) | -57 – -55 | -58 –  -54 | -58 –  -55 | -61 –  -60 | -59 –  -56 | -58 – -55 | -56 –  -54 | -57 – -56 | -58 –  -57 | -58 –  -56 | -59 – -58 | -61–  -57 | -57 | -57–  -55 |
| *δ*^18^OH_2_O (‰VSMOW) | -8.6 –-8.2 | -8.7 –-8.2 | -8.7– -8.5 | -9.0 –-8.8 | -8.8 – -8.5 | -8.4 –-8.3 | -8.4 –-8.2 | -8.6 –-8.3 | -8.8 – -8.3 | -8.8 –-8.3 | –8.7 - –8.6 | -8.8 –-8.6 | -8.6 –-8.5 | -8.6 –-8.3 |
| pH |  |  |  |  |  |  |  |  |  |  |  |  |  |  |
| 2007, Oct | 9.0 | 8.9 | 9.0 | 9.0 | 9.0 |  |  |  |  |  |  |  |  |  |
| 2009, Feb | 8.7 | 8.6 | 8.7 | 8.6 | 8.7 | 8.5 | 8.6 | 8.4 | 8.3 | 8.2 |  |  |  |  |
| 2011, Oct | 8.7 | 8.6 | 8.7 | 8.7 | 8.7 | 8.6 | 8.6 | 8.6 | 8.4 | 8.5 | 8.4 | 8.4 | 8.5 | 8.5 |
| 2012, Dec | 8.9 | 8.9 | 8.9 | 8.9 | 8.9 | 8.7 | 8.7 | 8.7 | 8.6 | 8.6 | 8.5 | 8.5 | 8.6 | 8.6 |
| Na^+^ (mM) |  |  |  |  |  |  |  |  |  |  |  |  |  |  |
| 2007, Oct | 3.3 | 3.7 | 3.8 | 4.4 | 4.8 |  |  |  |  |  |  |  |  |  |
| 2009, Feb | 3.3 | 3.6 | 3.8 | 4.2 | 4.2 | 3.3 | 3.3 | 4.4 | 5.9 | 6.5 |  |  |  |  |
| 2011, Oct | 3.2 | 3.7 | 4.0 | 4.6 | 4.6 | 3.5 | 3.4 | 3.6 | 4.3 | 3.9 | 5.1 | 5.2 | 4.1 | 4.6 |
| 2012, Dec | 3.2 | 3.7 | 4.3 | 4.7 | 4.7 | 3.6 | 3.5 | 3.7 | 4.4 | 4.3 | 4.9 | 4.6 | 3.8 | 3.7 |
| K^+^ (μM) |  |  |  |  |  |  |  |  |  |  |  |  |  |  |
| 2007, Oct | 10.2 | 12.8 | 17.9 | 12.8 | 20.5 |  |  |  |  |  |  |  |  |  |
| 2009, Feb | 10.2 | 10.2 | 10.2 | 12.8 | 12.8 | 7.7 | 7.7 | 15.3 | 20.5 | 25.6 |  |  |  |  |
| 2011, Oct | 7.7 | 10.2 | 10.2 | 12.8 | 12.8 | 10.2 | 10.2 | 10.2 | 17.9 | 15.3 | 20.5 | 20.5 | 15.3 | 15.3 |
| 2012, Dec | 7.7 | 10.2 | 12.8 | 12.8 | 12.8 | 10.2 | 10.2 | 12.8 | 17.9 | 17.9 | 17.9 | 17.9 | 15.3 | 12.8 |
| Ca^2+^ (mM) |  |  |  |  |  |  |  |  |  |  |  |  |  |  |
| 2007, Oct | 0.3 | 0.3 | 0.4 | 0.5 | 0.6 |  |  |  |  |  |  |  |  |  |
| 2009, Feb | 0.3 | 0.3 | 0.3 | 0.4 | 0.4 | 0.2 | 0.2 | 0.4 | 0.6 | 0.8 |  |  |  |  |
| 2011, Oct | 0.2 | 0.3 | 0.3 | 0.4 | 0.4 | 0.2 | 0.2 | 0.2 | 0.3 | 0.3 | 0.4 | 0.4 | 0.3 | 0.5 |
| 2012, Dec | 0.2 | 0.3 | 0.4 | 0.5 | 0.5 | 0.2 | 0.2 | 0.3 | 0.4 | 0.4 | 0.4 | 0.3 | 0.3 | 0.3 |
| Mg^2+^ (μM) |  |  |  |  |  |  |  |  |  |  |  |  |  |  |
| 2007, Oct | <4.1 | 4.1 | <4.1 | 4.1 | 4.1 |  |  |  |  |  |  |  |  |  |
| 2009, Feb | <4.1 | <4.1 | <4.1 | <4.1 | <4.1 | <4.1 | <4.1 | 12.3 | 49.4 | 53.5 |  |  |  |  |
| 2011, Oct | <4.1 | <4.1 | <4.1 | <4.1 | <4.1 | 4.1 | <4.1 | 12.3 | 28.8 | 20.6 | 28.8 | 32.9 | 20.6 | 16.5 |
| 2012, Dec | <4.1 | <4.1 | 4.1 | <4.1 | <4.1 | 4.1 | 4.1 | 12.3 | 32.9 | 28.8 | 28.8 | 24.7 | 20.6 | 12.3 |
| Cl^–^ (mM) |  |  |  |  |  |  |  |  |  |  |  |  |  |  |
| 2007, Oct | 1.8 | 2.5 | 2.7 | 4.0 | 4.6 |  |  |  |  |  |  |  |  |  |
| 2009, Feb | 1.7 | 2.1 | 2.5 | 3.3 | 3.4 | 1.6 | 1.5 | 3.4 | 5.8 | 7.0 |  |  |  |  |
| 2011, Oct | 1.3 | 2.2 | 2.9 | 4.1 | 4.1 | 1.7 | 1.6 | 2.1 | 3.0 | 2.4 | 4.4 | 4.4 | 3.0 | 4.1 |
| 2012, Dec | 1.4 | 2.5 | 3.5 | 4.3 | 4.4 | 2.0 | 1.8 | 2.2 | 3.2 | 3.3 | 4.0 | 3.2 | 2.3 | 2.3 |
| DIC (mM) |  |  |  |  |  |  |  |  |  |  |  |  |  |  |
| 2007, Oct | 1.2 | 0.9 | 0.8 | 0.6 | 0.5 |  |  |  |  |  |  |  |  |  |
| 2009, Feb | 1.1 | 0.9 | 0.7 | 0.7 | 0.6 | 1.2 | 1.2 | 1.0 | 0.9 | 0.7 |  |  |  |  |
| 2011, Oct | 1.4 | 1.2 | 1.0 | 0.8 | 0.8 | 1.3 | 1.3 | 1.3 | 1.3 | 1.3 | 1.1 | 1.1 | 1.1 | 0.9 |
| 2012, Dec | 1.3 | 1.0 | 0.8 | 0.6 | 0.6 | 1.2 | 1.2 | 1.2 | 1.3 | 1.1 | 1.2 | 1.3 | 1.3 | 1.2 |
| SI CaCO3 |  |  |  |  |  |  |  |  |  |  |  |  |  |  |
| 2007, Oct | -0.17 | -0.21 | -0.16 | -0.13 | -0.13 |  |  |  |  |  |  |  |  |  |
| 2009, Feb | -0.44 | -0.4 | -0.49 | -0.55 | -0.45 | -0.84 | -0.74 | -0.71 | -0.71 | -0.79 |  |  |  |  |
| 2011, Oct | -0.57 | -0.62 | -0.53 | -0.54 | -0.54 | -0.66 | -0.67 |  |  |  | -0.67 | -0.67 | -0.63 | -0.62 |
| 2012, Dec | -0.39 | -0.34 | -0.36 | -0.38 | -0.4 | -0.57 | -0.58 | -0.49 | -0.48 | -0.54 | -0.56 | -0.58 | -0.56 | -0.59 |
| SI CaSO4 |  |  |  |  |  |  |  |  |  |  |  |  |  |  |
| 2007, Oct | -3.07 | -3.06 | -3.12 | -3.25 | -3.29 |  |  |  |  |  |  |  |  |  |
| 2009, Feb | -3.03 | -2.91 | -3.04 | -3.06 | -3.08 | -3.15 | -3.15 | -2.97 | -3.17 | -3.05 |  |  |  |  |
| 2011, Oct | -3.12 | -3.08 | -3.09 | -3.17 | -3.18 | -3.15 | -3.15 |  |  |  | -3.26 | -3.28 | -3.12 | -2.98 |
| 2012, Dec | -3.13 | -3.09 | -3.17 | -3.28 | -3.3 | -3.19 | -3.16 | -3.12 | -3.12 | -3.08 | -3.21 | -3.11 | -3.05 | -3.02 |

Table S2. Groundwater concentrations of biologically utilized compounds.

|  | 07MI07-1 | 07MI07-2 | 07MI07-3 | 07MI07-4 | 07MI07-5 | 09MI20-1 | 09MI20–2 | 09MI20–3 | 09MI20–4 | 09MI20-5 | 10MI26-1 | 10MI26-3 | 10MI26-4 | 10MI26-5 |
| --- | --- | --- | --- | --- | --- | --- | --- | --- | --- | --- | --- | --- | --- | --- |
| DO (μM) | <0.6 | <0.6 | <0.6 | <0.6 | <0.6 | <0.6 | <0.6 | <0.6 | <0.6 | <0.6 | <0.6 | <0.6 | <0.6 | <0.6 |
| NH_4_^+^ (μM) | <11.2 | <11.2 | <11.2 | <11.2 | <11.2 | <11.2 | <11.2 | <11.2 | <11.2 | <11.2 | <11.2 | <11.2 | <11.2 | <11.2 |
| NO_3_^–^ (μM) | <0.8 | <0.8 | <0.8 | <0.8 | <0.8 | <0.8 | <0.8 | <0.8 | <0.8 | <0.8 | <0.8 | <0.8 | <0.8 | <0.8 |
| NO_2_^–^ (μM) | <1.1 | <1.1 | <1.1 | <1.1 | <1.1 | <1.1 | <1.1 | <1.1 | <1.1 | <1.1 | <1.1 | <1.1 | <1.1 | <1.1 |
| PO_4_^3–^ (μM) | <5.3 | <5.3 | <5.3 | <5.3 | <5.3 | <5.3 | <5.3 | <5.3 | <5.3 | <5.3 | <5.3 | <5.3 | <5.3 | <5.3 |
| Fe (μM) | <0.15 | <0.15 | <0.15 | <0.15 | <0.15 | <0.15 | <0.15 | <0.15 | <0.15 | <0.15 | <0.15 | <0.15 | <0.15 | <0.15 |
| Mn (μM) | <0.15 | <0.15 | <0.15 | <0.15 | <0.15 | <0.15 | <0.15 | <0.15 | <0.15 | <0.15 | <0.15 | <0.15 | <0.15 | <0.15 |
| DOC (μM) |  |  |  |  |  |  |  |  |  |  |  |  |  |  |
| 2007–2011 | <66.7 | <66.7 | <66.7 | <66.7 | <66.7 | <66.7 | <66.7 | <66.7 | <66.7 | <66.7 | <66.7 | <66.7 | <66.7 | <66.7 |
| 2011, Aug | 40.6 | 53.3 | 43.6 | 37 | 34.6 | 41.1 | 40.1 | 44.8 | 49.3 | 41 | 45.8 | 32.7 | 33.8 | 39.6 |
| 2012, Aug | 39.2 | 39.2 | 31.7 | 24.2 | 20.8 | 44.2 | 52.5 | 48.3 | 50 | 37.5 | 51.7 | 34.2 | 35.8 | 34.2 |
| Acetate (μM) |  |  |  |  |  |  |  |  |  |  |  |  |  |  |
| 2008–2011 | <2.7 | <2.7 | <2.7 | <2.7 | <2.7 | <2.7 | <2.7 | <2.7 | <2.7 | <2.7 | <2.7 | <2.7 | <2.7 | <2.7 |
| 2012, Aug | 3.3 | 4 | <2.7 | 3.5 | <2.7 | <2.7 | <2.7 | <2.7 | <2.7 | <2.7 | <2.7 | <2.7 | <2.7 | <2.7 |
| C_2_H_6_ (μM) |  |  |  |  |  |  |  |  |  |  |  |  |  |  |
| 2009, Sep | <0.1 | <0.1 | <0.1 | <0.1 | <0.1 | <0.1 | <0.1 | <0.1 | <0.1 | <0.1 |  |  |  |  |
| 2011, Feb | 61 |  | 88 |  | 55 | <0.1 |  | 155 |  | <0.1 | <0.1 | <0.1 | <0.1 | <0.1 |
| 2012, Jul | 8 | 7 | 15 | 20 | 21 | 9 | 9 | 11 | 16 | 15 | 20 | 17 | 13 | 14 |
| CH_4_ (μM) |  |  |  |  |  |  |  |  |  |  |  |  |  |  |
| 2009, Sep | 277 |  | 366 | 256 | 484 | 179 | 127 | 382 | 629 | 750 |  |  |  |  |
| 2011, Feb | 99 |  | 195 |  | 284 | 146 |  | 199 |  | 277 | 455 | 446 | 347 | 306 |
| 2012, Jul | 124 | 198 | 296 | 409 | 422 | 203 | 221 | 225 | 502 | 282 | 487 | 378 | 286 | 266 |
| HS^–^ (μM) |  |  |  |  |  |  |  |  |  |  |  |  |  |  |
| 2008, May | 3.4 | 4.4 | 3.4 | 4.1 | 11.6 |  |  |  |  |  |  |  |  |  |
| 2009, Nov | 21.9 | 21.9 | 21.9 | 25.0 | 25.0 | 25.0 | 25.0 | 18.8 | 3.1 | 3.1 |  |  |  |  |
| 2010, Oct | 18.8 |  | 15.6 |  | 18.8 | 12.5 |  | 9.4 |  | 6.3 | 6.3 | 6.3 | 12.5 | 12.5 |
| 2011, Feb |  |  |  |  |  |  |  |  |  |  | 6.3 | 6.3 | 9.4 | 9.4 |
| 2011, Aug | 18.8 |  |  |  |  | 12.5 | 18.8 |  | 6.3 |  |  |  |  |  |
| 2012, Aug | 21.9 | 21.9 | 21.9 | 25.0 | 21.9 | 21.9 | 21.9 | 9.4 | 6.3 | 6.3 | 3.1 | 6.3 | 9.4 | 12.5 |
| SO_4_^2–^ (μM) |  |  |  |  |  |  |  |  |  |  |  |  |  |  |
| 2008, May | 111 | 76 | 60 | 46 | 45 |  |  |  |  |  |  |  |  |  |
| 2009, Nov | 167 | 146 | 146 | 115 | 104 | 167 | 167 | 125 | 67 | 58 |  |  |  |  |
| 2010, Oct | 156 |  | 125 |  | 81 | 156 |  | 156 |  | 135 | 67 | 64 | 104 | 97 |
| 2011, Feb |  |  |  |  |  |  |  |  |  |  | 65 | 63 | 99 | 98 |
| 2011, Aug | 177 |  |  |  |  | 146 | 156 |  | 115 |  |  |  |  |  |
| 2012, Aug | 177 | 125 | 100 | 72 | 71 | 188 | 188 | 146 | 115 | 125 | 75 | 93 | 146 | 146 |
| H_2_ (nM) |  |  |  |  |  |  |  |  |  |  |  |  |  |  |
| 2010, July | 2.4 |  | 2.5 |  | 2 | 2.4 | 1.7 | 2.1 |  |  |  |  |  |  |
| 2011, Feb | 16.3 |  |  |  |  | 2.9 |  |  |  |  | 3.4 | 4.1 | 8.3 | 3.6 |
| 2011, Aug | 8.1 | 12.2 | 7.7 | 16.3 | 15.5 | 2.8 | 4.1 | 4 | 9.5 | 9.5 | 10.1 | 5.2 | 9.5 | 7.3 |
| 2012, July | 1.6 | 4.1 | 1.2 | 1.5 | 0.8 | 3.1 | 2.4 | 2.1 | 3.8 | 15.7 | 4.7 | 1.6 | 3 | 2.6 |

Table S3. Carbon, hydrogen and sulphur isotopic composition of biologically metabolized compounds in groundwater. VPDB and CDT indicate Vienna Pee Dee Belemnite and Cañon Diablo meteorite, respectively.

|  | 07MI07-1 | 07MI07-2 | 07MI07-3 | 07MI07-4 | 07MI07-5 | 09MI20-1 | 09MI20-2 | 09MI20-3 | 09MI20-4 | 09MI20-5 | 10MI26-1 | 10MI26-3 | 10MI26-4 | 10MI26-5 |
| --- | --- | --- | --- | --- | --- | --- | --- | --- | --- | --- | --- | --- | --- | --- |
| δ^13^C_CH4_ (‰VPDB) |  |  |  |  |  |  |  |  |  |  |  |  |  |  |
| 2009, Sep | –32.7 | –33.7 | –28.5 | –40.8 | –33.5 | –35.8 | –34.7 | –34.4 | –37.7 | –34.2 |  |  |  |  |
| 2011, Feb | –34.8 |  | –34.9 |  | –35.2 | –34.5 |  | –35.6 |  | –35.8 | –38.1 | –38.6 | –37.0 | –35.0 |
| 2012, Jul | –32.9 | –33.9 | –34.9 | –35.0 | –35.2 | –35.8 | –36.8 | –36.8 | –46.3 | –35.0 | –38.8 | –39.5 | –37.1 | –35.5 |
| δD_CH4_ (‰VSMOW) |  |  |  |  |  |  |  |  |  |  |  |  |  |  |
| 2009, Sep | –131 | –126 | –131 | –130 | –132 | –112 | –111 | –128 | –142 | –136 |  |  |  |  |
| 2011, Feb | –107 |  | –122 |  | –127 | –122 |  | –138 |  | –140 | –148 | –153 | –141 | –141 |
| 2012, Jul | –94 | –108 | –127 | –129 | –129 | –110 | –118 | –125 | –231 | –125 | –143 | –142 | –128 | –121 |
| δ^13^C_DIC_ (‰VPDB) |  |  |  |  |  |  |  |  |  |  |  |  |  |  |
| 2007, Nov | –13.7 | –13.7 | –13.8 | –13.9 | –13.3 |  |  |  |  |  |  |  |  |  |
| 2008, Jul | –16.1 | –12.4 | –12.4 | –12.4 | –12.5 |  |  |  |  |  |  |  |  |  |
| 2009, Nov | –16.7 | –15.2 | –14.8 | –15.3 | –15.3 | –15.4 | –12.9 | –8.9 | –10.0 |  |  |  |  |  |
| 2010, Aug | –15.7 | –15.9 | –15.6 | –15.8 | –15.5 | –15.6 | –13.8 | –9.7 | –11.3 |  |  |  |  |  |
| 2011, Aug | –16 | –17.3 | –17.9 | –15.8 | –15.9 | –14.6 | –13.1 | –20.7 | –12.6 | –8.2 | –9.8 | –11.4 |  |  |
| 2012, Jun | –17 | –16.0 | –15.8 | –15.6 | –15.9 | –15.6 | –14.6 | –9.8 | –12.3 | –8.9 | –12.0 | –12.8 |  |  |
| δ^34^S_HS–_ (‰CDT) |  |  |  |  |  |  |  |  |  |  |  |  |  |  |
| 2008, May | –34.5 | –33.2 | –21.4 | –21.0 | –8.8 |  |  |  |  |  |  |  |  |  |
| 2009, Nov | –39.5 | –34.0 | –32.5 | –24.9 | –23.7 | –41.3 | –41.5 | –31.5 | –24.9 | –17.5 |  |  |  |  |
| 2010, Oct | –42.2 |  | –31.7 |  | –19.3 | –43.0 |  | –31.4 |  | –30.2 |  |  |  |  |
| 2011, Feb |  |  |  |  |  |  |  |  |  |  | 3.9 | 5.4 | –6.9 | –6.4 |
| 2011, Aug | –20.0 |  |  |  |  | –19.1 | –18.2 |  | –2.8 |  | 2.8 | 5.9 | –1.9 | –6.0 |
| 2012, Aug | –15.7 | –14.0 | –7.2 | –1.7 | –2.0 | –14.6 | –16.9 | –9.2 | –2.1 | –2.5 | 8.5 | 1.8 | –4.3 | –7.7 |
| δ^34^S_SO4_ (‰CDT) |  |  |  |  |  |  |  |  |  |  |  |  |  |  |
| 2008, May | 9.3 | 8.2 | 16.6 | 16.2 | 29.9 |  |  |  |  |  |  |  |  |  |
| 2009, Nov | 11.4 | 13.6 | 14.4 | 17.9 | 18.9 | 11.9 | 11.5 | 16.2 | 21.4 | 24.2 |  |  |  |  |
| 2010, Oct | 12.1 |  | 16.1 |  | 24.0 | 10.9 |  | 12.3 |  | 13.9 |  |  |  |  |
| 2011, Feb |  |  |  |  |  |  |  |  |  |  | 26.4 | 24.6 | 17.5 | 18.8 |
| 2011, Aug | 9.8 |  |  |  |  | 12.9 | 12.0 |  | 16.6 |  | 25.5 | 26.5 | 19.8 | 19.6 |
| 2012, Aug | 12.4 | 13.0 | 13.0 | 31.7 | 34.1 | 12.9 | 10.4 | 16.1 | 16.8 | 17.3 | 26.7 | 22.1 | 16.5 | 16.0 |
| *Δ*^34^S_SO42—HS-_  (‰CDT) |  |  |  |  |  |  |  |  |  |  |  |  |  |  |
| 2008, May | 43.7 | 41.5 | 38.0 | 37.2 | 38.7 |  |  |  |  |  |  |  |  |  |
| 2009, Nov | 50.8 | 47.6 | 46.9 | 42.8 | 42.7 | 53.2 | 53.0 | 47.6 | 46.3 | 41.7 |  |  |  |  |
| 2010, Oct | 54.3 |  | 47.9 |  | 43.3 | 53.9 |  | 43.7 |  | 44.1 |  |  |  |  |
| 2011, Feb |  |  |  |  |  |  |  |  |  |  | 22.5 | 19.2 | 24.4 | 25.2 |
| 2011, Aug | 29.8 |  |  |  |  | 32.0 | 30.2 |  | 19.4 |  | 22.7 | 20.7 | 21.7 | 25.6 |
| 2012, Aug | 28.1 | 27.0 | 20.2 | 33.4 | 36.1 | 27.5 | 27.3 | 25.3 | 18.9 | 19.8 | 18.2 | 20.3 | 20.8 | 23.7 |
| δ^13^C_DIC_ (‰VPDB) |  |  |  |  |  |  |  |  |  |  |  |  |  |  |
| 2007, Nov | –13.7 | –13.7 | –13.8 | –13.9 | –13.3 |  |  |  |  |  |  |  |  |  |
| 2008, Jul | –16.1 | –12.4 | –12.4 | –12.4 | –12.5 |  |  |  |  |  |  |  |  |  |
| 2009, Nov | –16.7 | –15.2 | –14.8 | –15.3 | –15.3 | –15.0 | –15.4 | –12.9 | –8.9 | –10.0 |  |  |  |  |
| 2010, Aug | –15.7 | –15.9 | –15.6 | –15.8 | –15.5 | –15.6 | –15.6 | –13.8 | –9.7 | –11.3 |  |  |  |  |
| 2011, Aug | –16 | –17.3 | –17.9 | –15.8 | –15.9 | –14.3 | –14.6 | –13.1 | –20.7 | –12.6 | –8.2 | –7.5 | –9.8 | –11.4 |
| 2012, Jun | –17 | –16.0 | –15.8 | –15.6 | –15.9 | –15.5 | –15.6 | –14.6 | –9.8 | –12.3 | –8.9 | –8.4 | –12.0 | –12.8 |

Table S4. Groundwater concentrations and isotopic composition of noble gases.

|  | ^3^He (10^–11^cm^3^STP/gH_2_O) | ^4^He (10^–5^cm^3^STP/gH_2_O) | ^20^Ne  (10^–7^cm^3^STP/gH_2_O) | ^3^He/^4^He (10^–6^) | ^4^He/^20^Ne |
| --- | --- | --- | --- | --- | --- |
| 09MI20-1 (2012, Nov) | 2.78 | 6.35 | 4.23 | 0.454 ± 0.005 | 150 |
| 09MI20-3 (2012, Nov) | 2.14 | 4.65 | 3.00 | 0.460 ± 0.007 | 155 |
| 09MI26-5 (2012, Nov) | 1.99 | 4.03 | 2.94 | 0.493 ± 0.011 | 137 |

Table S5. Estimated amounts of microbially reduced sulphate in the granitic aquifer.

|  | 07MI07- 1 | 07MI07-2 | 07MI07-3 | 07MI07-4 | 07MI07-5 | 09MI20-1 | 09MI20-2 | 09MI20-3 | 09MI20-4 | 09MI20-5 |
| --- | --- | --- | --- | --- | --- | --- | --- | --- | --- | --- |
| 2008, May (mM) | –1.7 | –2.8 | 8.6 | 6.3 | 19.6 |  |  |  |  |  |
| 2009, Nov  (mM) | 4.4 | 10.4 | 12.6 | 18.5 | 19.2 | 6.1 | 4.8 | 15.6 | 16.3 | 18.0 |
| 2010, Oct  (mM) | 6.5 |  | 15.5 |  | 24.0 | 2.8 |  | 7.0 |  | 10.7 |

Supplementary Figure 1


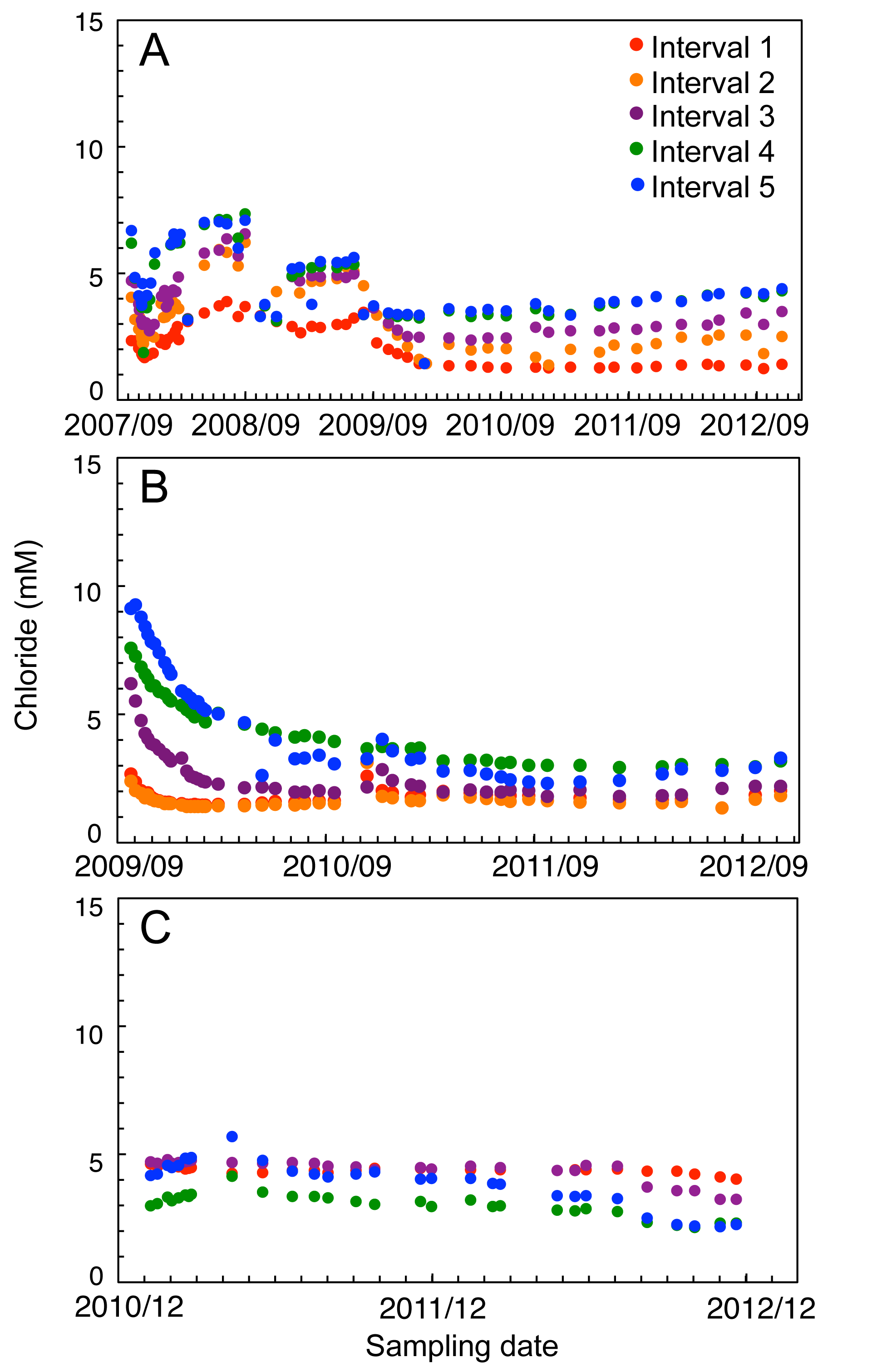


Supplementary Figure 2


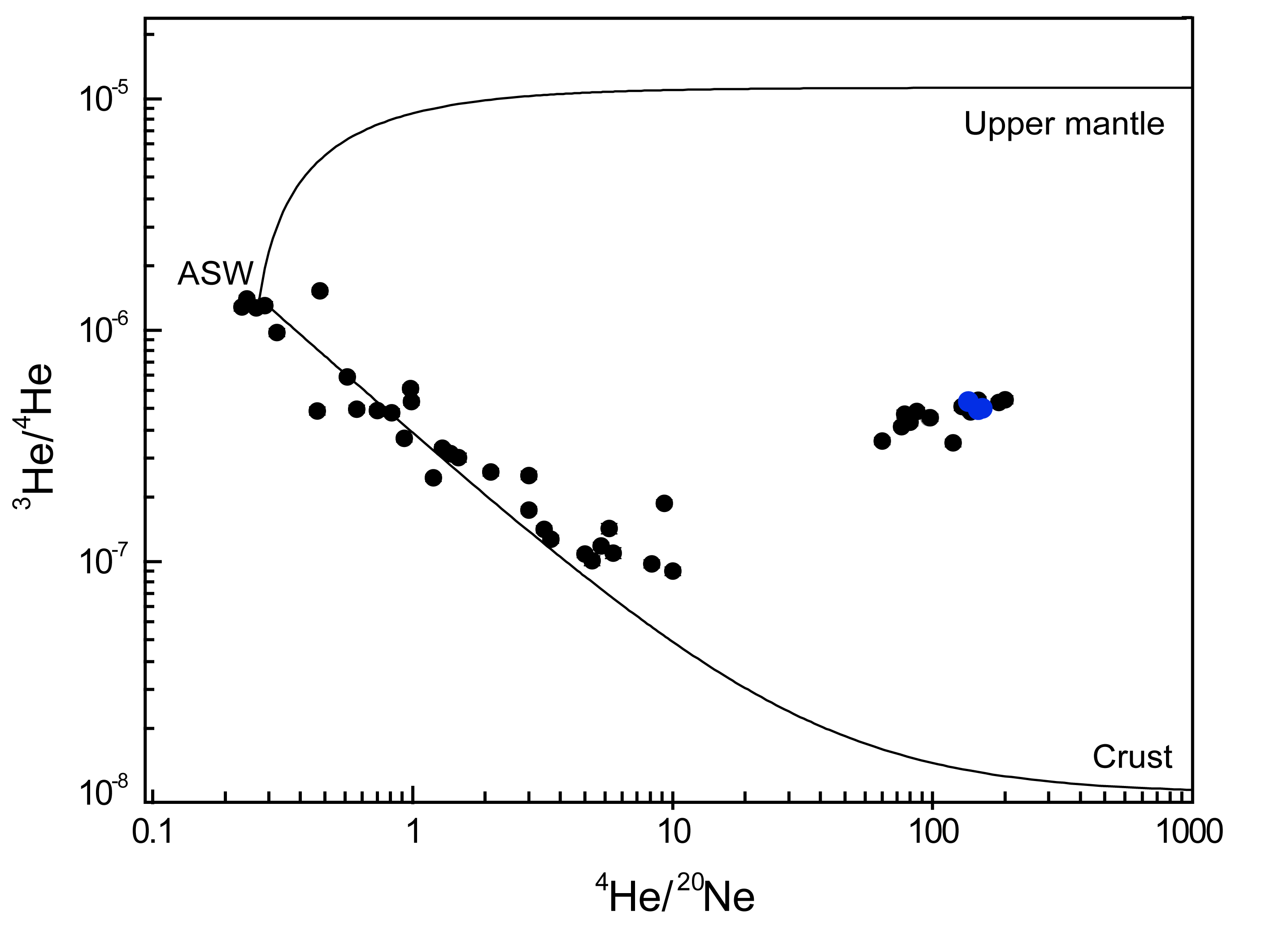

Supplement: S1 File — S1 Figure, Changes in chloride concentration during shaft construction: (A) 07MI07 borehole, (B) 09MI20 borehole, (C) 10MI26 borehole. Each circle colour represents different sampling intervals. S2 Figure, Correlation between 3He/4He and 4He/20Ne in deep groundwater samples from boreholes drilled from the surface in the Tono area (black circles) [22] and from the MIU sub-stages (blue circles). The solid lines delineate the mixing lines between air saturated water (ASW) and the upper mantle, and between ASW and the crust. S1 Table, Temperature, isotopic composition and pH of groundwater samples and the concentrations of major cations and anions as well as saturation indices (SI) of calcium carbonate and sulphate minerals. VSMOW = Vienna Standard Mean Ocean Water. S2 Table, Groundwater concentrations of biologically utilized compounds. S3 Table, Carbon, hydrogen and sulphur isotopic composition of biologically metabolized compounds in groundwater. VPDB and CDT indicate Vienna Pee Dee Belemnite and Cañon Diablo meteorite, respectively. S4 Table, Groundwater concentrations and isotopic composition of noble gases. S5 Table, Estimated amounts of microbially reduced sulphate in the granitic aquifer. (DOCX) [file pone.0113063.s001.docx]
